# Association between serum potassium and risk of all‐cause mortality among chronic kidney diseases patients: A systematic review and dose–response meta‐analysis of more than one million participants

**DOI:** 10.1002/fsn3.2478

**Published:** 2021-07-23

**Authors:** Nasim Sorraya, Amirhosein Farrokhzad, Bahar Hassani, Shokoofeh Talebi

**Affiliations:** ^1^ Department of Community Nutrition School of Nutrition and Food Science Food Security Research Center Isfahan University of Medical Sciences Isfahan Iran; ^2^ Department of Orthopedics Isfahan University of Medical Sciences Isfahan Iran; ^3^ Department of Nutrition Ahvaz Jundishapur University of Medical Sciences Ahvaz Iran; ^4^ Department of Health Safety and Environment (HSE) Razi Petrochemical Company Mahshahr Iran; ^5^ Department of Clinical Nutrition School of Nutrition and Food Science Food Security Research Center Isfahan University of Medical Sciences Isfahan Iran

**Keywords:** chronic kidney disease, dose, meta‐analysis, mortality, potassium, response

## Abstract

We aimed to perform a meta‐analysis, using prospective cohort studies, to test the association between serum potassium and all‐cause mortality among chronic kidney diseases (CKD) patients. A systematic search was performed using PubMed‐MEDLINE and Scopus, up to July 2020. Prospective cohort studies which reported risk estimates of all‐cause mortality in CKD patients with different serum potassium levels were included in the present meta‐analysis. Thirteen studies were included in the analysis. A nonlinear dose–response meta‐analysis suggested that there is a J‐shaped association between serum potassium levels and the risk of all‐cause mortality, with a nadir at serum potassium of 4.5 mmol/L. Subgroup analyses indicated that the strength and shape of the association between serum potassium and all‐cause mortality may be influenced by age. Our meta‐analysis provides supportive evidence that there is a J‐shape association between serum potassium and all‐cause mortality among CKD patients.

## Graphical abstract

Potassium disorders or dyskalemias (hypo‐ and hyperkalemia) have been associated with mortality in cardiovascular diseases and renal patients. There has been no meta‐analysis to summarize the association between serum potassium levels and mortality among CKD patients. Present meta‐analysis provides supportive evidence that there is a J‐shape association between serum potassium and all‐cause mortality among CKD patients.

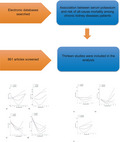

## 1 INTRODUCTION

Chronic kidney disease (CKD) is an emerging major public health concern worldwide because of its rising incidence and prevalence, high risk of progression to end‐stage renal disease (ESRD), and poor prognosis (El Nahas & Bello, 2005; Levey et al., 2007). CKD is diagnosed when there is an evidence of kidney damage lasting for more than three months. Based on the estimated glomerular filtration rate (GFR), CKD is divided into five stages (Evans & Taal, 2011). The number of patients with CKD is anticipated to rise in the near future. The prevalence of CKD in developed countries such as the United States ranges from 13% to16% (Coresh et al., 2007; L. Zhang et al., 2008). In developing countries, CKD prevalence varies from 2% to 16% (Ito et al., 2008; Singh et al., 2009; Sumaili et al., 2008). Previous studies have reported an overall increase in CKD prevalence of about 5% per year in the US population (Grams et al., 2013; Hsu & Hsu, 2013). The increasing prevalence of CKD could be related to the ageing of the population, the age‐related decline in renal function and the rapidly increasing incidence of diabetes and hypertension (McFadden et al., 2018; Singh et al., 2013).

Despite the advances in the treatment of CKD, mortality remains high, especially in patients with CKD undergoing dialysis (Evans et al., 2005; de Jager et al., 2009). The high mortality in dialysis‐treated patients is mostly related to the presence of cardiovascular diseases (CVD) (Himmelfarb & Ikizler, 2010). However, classical risk factors for CVD are relatively different in CKD patients compared to the general population. In CKD patients, a higher BMI and higher serum lipids levels are linked to a better survival (finding referred to as the “reverse epidemiology” phenomenon) (Moradi et al., 2015; de Mutsert et al., 2009; Rahimlu et al., 2017; Stenvinkel et al., 2016). Thus, finding possible predictive risk factors among CKD patients has received much attention recently.

Potassium disorders or dyskalemias (hypo‐ and hyperkalemia) have been associated with serious cardiac arrhythmias, CKD progression, ESRD, and mortality in CVD and renal patients (Collins et al., 2017; Wang et al., 2016). In CKD patients, there is a decline in the capacity to excrete potassium. Moreover, several medications may cause hypo‐ and hyperkalemia in these patients (Luo et al., 2016; Palmer, 2004). Hypokalemia has been associated with a higher mortality in dialysis patients because of its electrophysiological effects and its relationship with a poor nutritional status (Lowrie & Lew, 1990). On the other hand, mild reductions in serum K^+^ (3.5–<4.0 mEq/L) have been associated with excess mortality in patients with heart failure and/or CKD (Ahmed et al., 2007; Bowling et al., 2010; Wang et al., 2013). Hyperkalemia has been associated with a higher mortality because of the arrhythmogenic effects of potassium (Genovesi et al., 2009). Recent evidence suggests that both hypo‐ and hyperkalemia are associated with an increase in mortality (Hayes et al., 2012; Korgaonkar et al., 2010). However, the data are heterogeneous in terms of follow‐up duration and the number of potassium values per patient (Sinha & Agarwal, 2013).

To our knowledge, according to previously published studies, the association between serum K^+^ and CKD is inconsistent. Moreover, the association between serum potassium levels and mortality among CKD patients has not been evaluated systematically so far. Therefore, we aimed to conduct a systematic review and dose–response meta‐analysis, using prospective cohort studies, to test the linear and nonlinear dose–response association between serum potassium and all‐cause mortality among CKD patients. To the best of our knowledge, this is the first systematic review and meta‐analysis to examine this potential nonlinear dose–response relation.

## 2 METHODS

The Meta‐analysis of Observational Studies in Epidemiology (MOOSE) and the Preferred Reporting Items for Systematic Reviews and Meta‐analyses (PRISMA) items was employed in the implementation of this report.

### 2.1 Search strategy

A systematic search was performed using the PubMed‐MEDLINE and Scopus databases in order to collect relevant studies from inception up to July 2020. The following terms were used to search the databases: Title‐Abstract: [("Potassium") AND (“Mortality" OR "Death" OR "Deaths" OR "fatal" OR "Survival") AND ("Chronic kidney disorder" OR "CKD" OR "End stage renal disease" OR "Kidney Failure, Chronic" OR "ESRD" OR "renal failure" OR "Kidney Diseases" OR "Chronic renal insufficiency" OR "Renal Dialysis" OR "hemodialysis" OR "haemodialysis" OR "Dialysis" OR "Peritoneal Dialysis") AND ("prospective" OR "longitudinal" OR "follow‐up" OR "cohort" OR "retrospective")]. The search was restricted to articles published in English.

#### 2.1.1 Eligibility and study selection

Two independent reviewers (N.S. and Sh.T.) reviewed the titles and abstracts of all the studies. Prospective cohort studies were included in the present meta‐analysis. The studies were eligible if they: (a) were designed as a prospective cohort study, (b) were conducted in CKD patients aged more than 18 years, (c) had baseline measurements of potassium serum levels in patients with CKD, (d) had a minimum follow‐up of 1 year, (e) reported risk estimates (relative risk [RR] or hazard ratio [HR] or odds ratio) along with 95% confidence intervals (CI), (f) reported the mortality rate and number of participants for three and more quantitative subcategories, and (g) reported the number of cases and participants/person‐years in each category of serum potassium level or reported sufficient information to estimate those numbers. Studies that reported results per unit increment were also included. Studies were excluded if (a) they were designed as retrospective cohorts, cross‐sectional or case–control studies; (b) they had only two categories of exposures; (c) they were conducted in children and adolescents; and (d) if the increase in serum potassium in continuous/linear form was not exactly identified.

#### 2.1.2 Data abstraction and quality assessment

Two reviewers (N.S. and Sh.T.) independently evaluated the full‐text of the selected articles and extracted the following data: first author's name, publication year, region of the study, age, gender, study population, follow‐up duration, number of participants and cases, RR and 95% CI of potassium serum levels, covariates adjusted in multivariate analysis. If in a given study results were reported as several adjustment models, the model with the most covariates adjustment was selected and used. Any discrepancies between authors were resolved by discussion and consultation. The quality of the included studies was evaluated by the Newcastle–Ottawa Scale (NOS) and studies with a score of 6 or higher were considered as high‐quality studies. Any discrepancies were resolved through discussion under the supervision of a third author (P.SH.).

#### 2.1.3 Data synthesis and Statistical analysis

We conducted a restricted cubic spline regression model to explore the possible nonlinear relation between serum potassium levels and mortality. When the lowest group was not the reference, HRs and CIs were recalculated relating to the referent for which data were required (Hamling et al., 2008). In addition, the midpoint of each serum potassium category was used if serum potassium means or median for the category was not reported in the study. If the highest or lowest categories were open‐ended categories, they were considered to have the same amplitude as their neighboring categories (this method was used for all studies with categorical variables) (Greenland & Longnecker, 1992; Orsini et al., 2006). If studies reported results for men and women or other subgroups separately, the subgroup‐specific estimates were combined by fixed‐effects model to produce an overall estimate and each study was only represented once in the final analysis. A two‐stage hierarchical regression model was conducted to calculate the nonlinear dose–response relation across different serum potassium consecrations. In this model, the difference between the medians of category‐specific and reference‐specific quadratic expressions for serum potassium was surveyed based on studies with nonzero serum potassium level as reference. The two‐stage generalized least squares trend estimation method was used, which first estimated study‐specific slope lines and then combined with studies in which the slopes were directly reported to acquire an overall average slope (Orsini et al., 2006). Then, the dose–response association, assuming within‐ and between‐study variances, was assessed using spline transformations. Potential nonlinear association was examined using restricted cubic splines with knots at fixed percentiles of the distribution (Orsini et al., 2011). A *p*‐value for nonlinearity of the meta‐analysis was estimated by testing the null hypothesis that the coefficient of the second spline was equal to zero. Moreover, the logarithms of HRs and CIs, the number of mortality and the number of participants across serum potassium categories were used to estimate linearity in the potential relationships by random‐effects dose–response (Liu et al., 2009). In linear dose–response the HRs related to 0.5 mmol/L increases in serum potassium for each study was estimated (Orsini et al., 2006). If two different articles reported results of the same study as categorical and continuous separately, the article with the categorical model was included in both linear and nonlinear dose–response meta‐analyses. The heterogeneity between studies was assessed by Q and I^2^ tests (*p* <.05), which provide the relative amount of variance of the summary effect due to the between‐study heterogeneity (Higgins et al., 2003). The Egger's test of asymmetry and funnel plot asymmetry test was used to evaluate any existing publication bias (Egger et al., 1997). In order to assess the impact of excluding one single study on overall RR estimation, sensitivity analysis was performed. All analyses were performed with the STATA software, version 14 (Stata Corp, College Station, Texas). *p*‐values <.05 were considered as significant.

## 3 RESULTS

### 3.1 Findings from the systematic review

**FIGURE 1:**
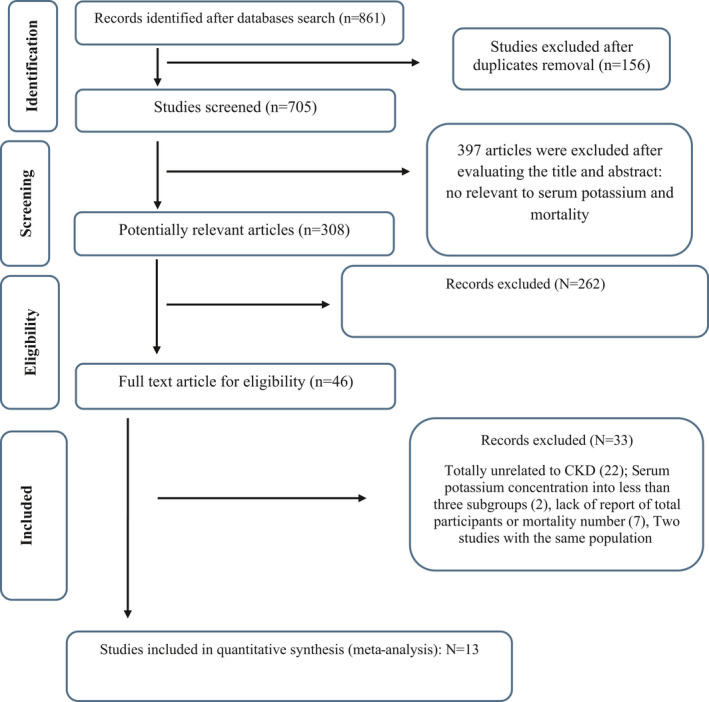
The procedure of study selection

Letters, comments, conference abstracts, short communications, animal studies, reviews, and meta‐analyses were excluded. Of the 861 articles identified from PubMed‐MEDLINE and Scopus in our initial search, 156 duplicate articles were removed. Authors surveyed the title‐abstract and where necessary the full‐texts of the included studies. Based on the initial title and abstract screening, 397 studies were excluded. Out of the 308 remaining articles, we excluded 262 other studies also due to different reasons: dietary potassium intake and mortality in long‐term CKD patients (Noori et al., 2010), dialysate potassium and risk of death in chronic hemodialysis (HD) patients (Adam, 2013; Al‐Ghamdi et al., 2010; Huang et al., 2015), urinary potassium excretion and mortality (Eisenga et al., 2016; He et al., 2016; Leonberg‐Yoo et al., 2017), potassium gradient categories and mortality (Brunelli et al., 2017), effect of different drugs on serum potassium levels (Bragg‐Gresham et al., 2007; Chan et al., 2010; Heerspink et al., 2014). Moreover, 22 studies were also excluded because they were not conducted in CKD patients and one study investigated Paraquat poisoning. Other studies were also excluded because they could not be used for the last dose‐response analysis: two studies were excluded because they did not report at least three categories of serum potassium for the reported RR (Abdulkader et al., 2003; An et al., 2012), others because the number of cases was not reported and the studies could not be used in the dose–response analysis (Abdulkader et al., 2003; Al‐Ghamdi et al., 2010; Chen et al., 2017; Collins et al., 2017; Karaboyas et al., 2017; Nakhoul et al., 2015; Ribeiro et al., 2015; Wagner et al., 2017; Yusuf et al., 2016). Of the remaining studies, two reported data on the same population (Hwang et al., 2013; Hwang et al., 2011). Finally, 13 studies matched our specified criteria and were used in the nonlinear and linear analysis (Einhorn et al., 2009; Flueckiger et al., 2014; Fukui et al., 2014; Hwang et al., 2013; Korgaonkar et al., 2010; Kovesdy et al., 2007; Lee et al., 2017; Li et al., 2015; Luo et al., 2016; Torlen et al., 2012; Xu et al., 2014; Zhang et al., 2016). Flow‐chart of the identification and selection process is shown in Figure 1. Included studies were conducted between 2001 and 2017. A total number of 1,122,325 participants and 123,274 cases were analyzed in the present study. The sample size of the studies ranged from 423 (Hwang et al., 2011) to 465,128 participants (Einhorn et al., 2009). The mean age of the participants ranged from 34 to 74 years. Patients’ follow‐up varied from 2 to 5 years. All of the included studies enrolled participants from both genders. The studies were performed in different countries: 11 in the United States (Einhorn et al., 2009; Flueckiger et al., 2014; S. Korgaonkar et al., 2010; Kovesdy et al., 2007; Lee et al., 2017; Luo et al., 2016; Torlen et al., 2012), three in China (Xu et al., 2014; Zhang et al., 2016), one in Korea (Lee et al., 2017), and one in Taiwan (Hwang et al., 2011). The average quality score of the studies was 7.7 (based on the NOS) (Stang, 2010). Serum potassium was measured during patients’ follow‐up with defined intervals. The characteristics of the included cohorts are outlined in Table 1. As depicted in Table 1, it must be noted that in some cases like Kim *et al*. (Lee et al., 2017), Torlen et al. (2012), or Einhorn et al. (2009), presented data for two arms; different races, peritoneal or HD and the inpatient and outpatient category, respectively. Therefore, the number of studies which has been included in the last dose–response analysis was based on the RR which has been presented.

**TABLE 1:** Characteristics of included cohorts

First author	Country	Study population	Follow‐up years	Mean Age	Gender	Participants (*n*)	Cases (*n*)	Reported HR or RR and 95% CI	NOS score	
Kovesdy C 2007	US	Hemodialysis	3	62	Both	81,013	22,075	1.45 (1.24 – 1.67)	9
EinhornLM 2009	US	Inpatient	1	73	Both	465,128	2,259	14.12 (13.3–15.06)	7	
EinhornLM 2009	US	Outpatient	1	73	Both	296,854	351	13.19 (12–14.66)	7	
Korgaongar S 2010	US	CKD	2.6	60	Both	720	86	0.9 (0.76 – 1.08)	8	
Hwang JC 2011	Taiwan	Hemodialysis	4	58	Both	423	90	0.55 (0.29 – 1.04)	6	
Torlen K 2012	US	Hemodialysis	5	62	Both	111,651	60,454	0.9 (0.85 – 0.93)	8	
Torlen K 2012	US	Peritoneal dialysis	5	56	Both	10,468	4,737	1.12 (1.09 – 1.15)	8	
Xu Q 2014	China	Peritoneal dialysis	3	50	Both	882	168	0.71 (0.56 – 0.98)	7	
Flueckiger P 2014	US	ESRD	3	50	Both	930	499	1.28 (0.96 – 1.70)	7	
Fukui M 2014	Japan	CKD	5.7	44	Both	1,001	263	0.31 (0.21 – 0.45)	8	
Li SH 2015	China	Peritoneal dialysis	2	50	Both	467	84	0.44 (0.17 – 1.11)	6	
Luo J 2016	US	CKD	4	74	Both	55,266	4,539	1.08 (0.99 – 1.17)	8	
Zhang YF 2016	China	ESRD	5	53	Both	676	222	0.78 (0.89 – 1.0)	8	
Lee S 2017	Korea	Dialysis	5	60	Both	3,230	751	0.07 (0.05 – 0.09)	9	
Kim T (1) 2017	US	Hemodialysis (white)	4	67	Both	51,297	16,804	1.17 (1.13 – 1.2)	8	
Kim T (2) 2017	US	Hemodialysis	4	61	Both	34,574	7,573	1.33 (1.03 – 1.119)	8	
Kim T (3) 2017	US	(African‐American)	4	54	Both	16,370	3,084	0.74 (0.73 – 0.75)	8	

Abbreviations: CKD, chronic kidney disease; ESRD, end‐stage renal disease.

### 3.2 Meta‐analysis findings

#### 3.2.1 Overall dose–response association between serum potassium and all‐cause mortality

**FIGURE 2:**
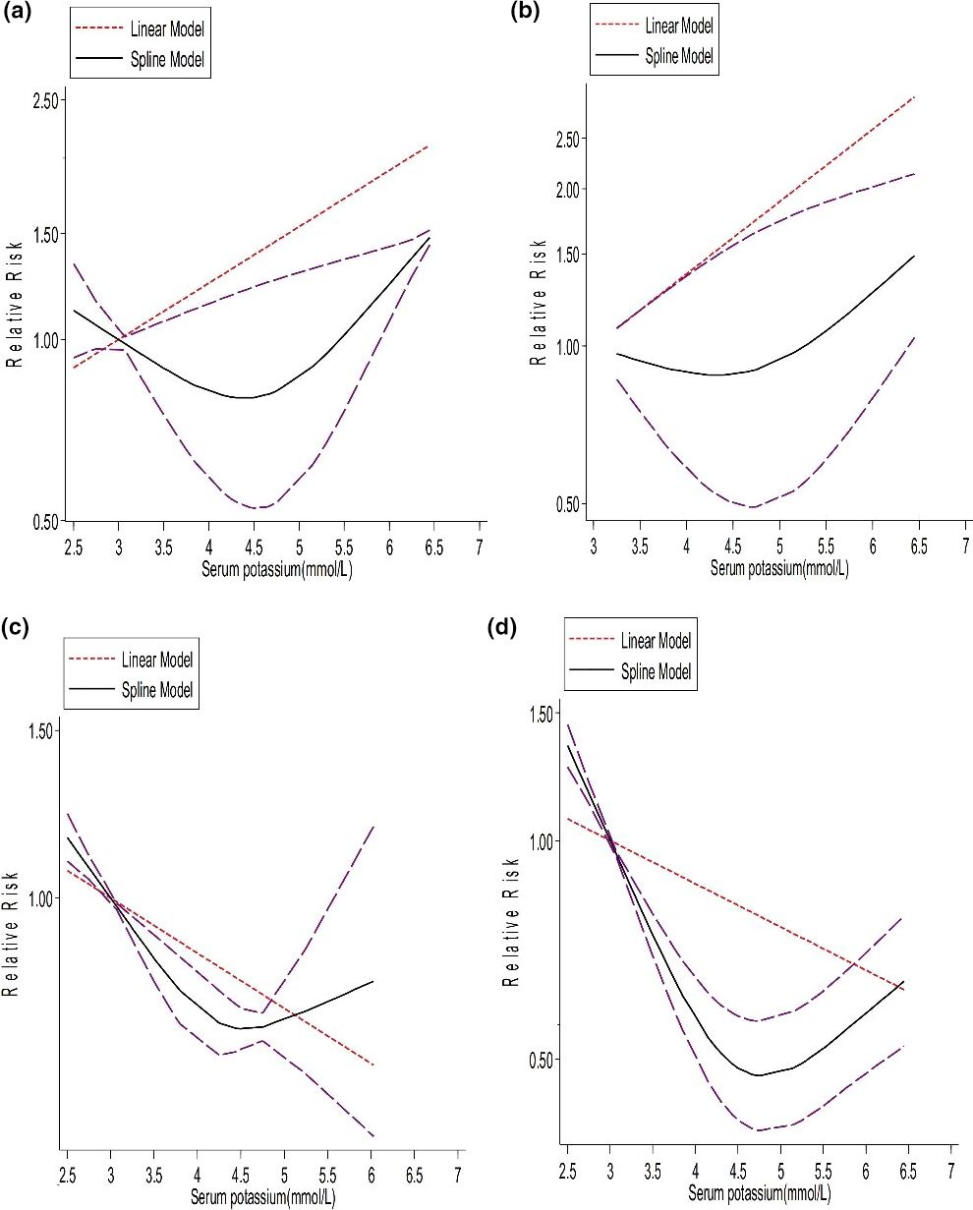
Horizontal axis shows serum potassium level and vertical axis shows OR. Solid line and long‐dash lines represent relative risk and 95% CIs. (a) Nonlinear dose–response association between serum potassium and risk of all‐cause mortality in chronic kidney disease (CKD) patients (whole analysis) *p* <.0001. (b) Nonlinear dose–response association between serum potassium and risk of all‐cause mortality in CKD patients (age of ≥60 years ‐ *p* <.0001). (c) Nonlinear dose–response association between serum potassium and risk of all‐cause mortality in CKD patients (age of <60 years – *p* <.0001). (d) Nonlinear dose–response association between serum potassium and risk of all‐cause mortality in CKD patients (with the exclusion of a large retrospective cohort study; *p* =.017)

Thirteen studies reported satisfactory information to test the possible nonlinear association (Einhorn et al., 2009; Heerspink et al., 2014; Hwang et al., 2011; Korgaonkar et al., 2010; Kovesdy et al., 2007; Lee et al., 2017; Li et al., 2015; J. Luo et al., 2016; Torlen et al., 2012; Xu et al., 2014; Zhang et al., 2016). The meta‐analysis indicated strong evidence of departure from linearity (*p* for nonlinearity < .0001, Figure 2a) with an estimate in the correlation matrix of 0.215 and an estimated between studies standard deviations (SDs) of 0.132 and 0.298 (Prob> chi^2^= 0.0000). The nonlinear dose–response meta‐analysis showed a J‐shaped association between serum potassium and the risk of all‐cause mortality in patients with CKD, with a nadir at serum potassium of 4.5 mmol/L. Serum potassium increasing up to 4.5 mmol/L had a protective effect on all‐cause mortality rates, but serum potassium more than 4.5 mmol/L in CKD patients lead to increase in the risk of mortality with a steep slope (Figure 2a).

#### 3.2.2 Subgroup analysis of dose–response association between serum potassium and all‐cause mortality

In addition, to test the potential effects of age on the association between serum potassium and all‐cause mortality in CKD, we then tested the dose–response relations across age subgroups (mean age >60 years versus. <60 years). Our results show that in CKD patients aged >60 years there is a J‐shaped association which was also statistically significant. Moreover, the risk of all‐cause mortality increased with the increase in serum potassium from a baseline of 4.5 mmol/L (*P* for nonlinearity <0.0001, *n* = 10 studies; Figure 2b). On the other hand, different association was seen in CKD patients with a mean age <60 years, with a nadir at serum potassium of 4.5 mmol/L do not follow J‐shape association (*p* for nonlinearity = 0.017, *n* = 6 studies; Figure 2c).

#### 3.2.3 Sensitivity analysis of dose–response association between serum potassium and all‐cause mortality

To test whether the obtained results could have been influenced by a large cohort study (Einhorn et al., 2009), a sensitivity analysis was performed by removing this study, and we found that the risk of mortality decreased continuously with increasing serum potassium up to about 4.8 mmol/L with a steep slope, and then increased with the same slope on the whole analysis (*p* for nonlinearity < 0.0001, estimate in the correlation matrix of −0.137 and the estimated between studies SDs of −0.19 and 0.83 (Prob> chi^2^= 0.0000) (Figure 2d).

#### 3.2.4 Dose–response association between serum potassium and all‐cause mortality in different CKD patients

**FIGURE 3:**
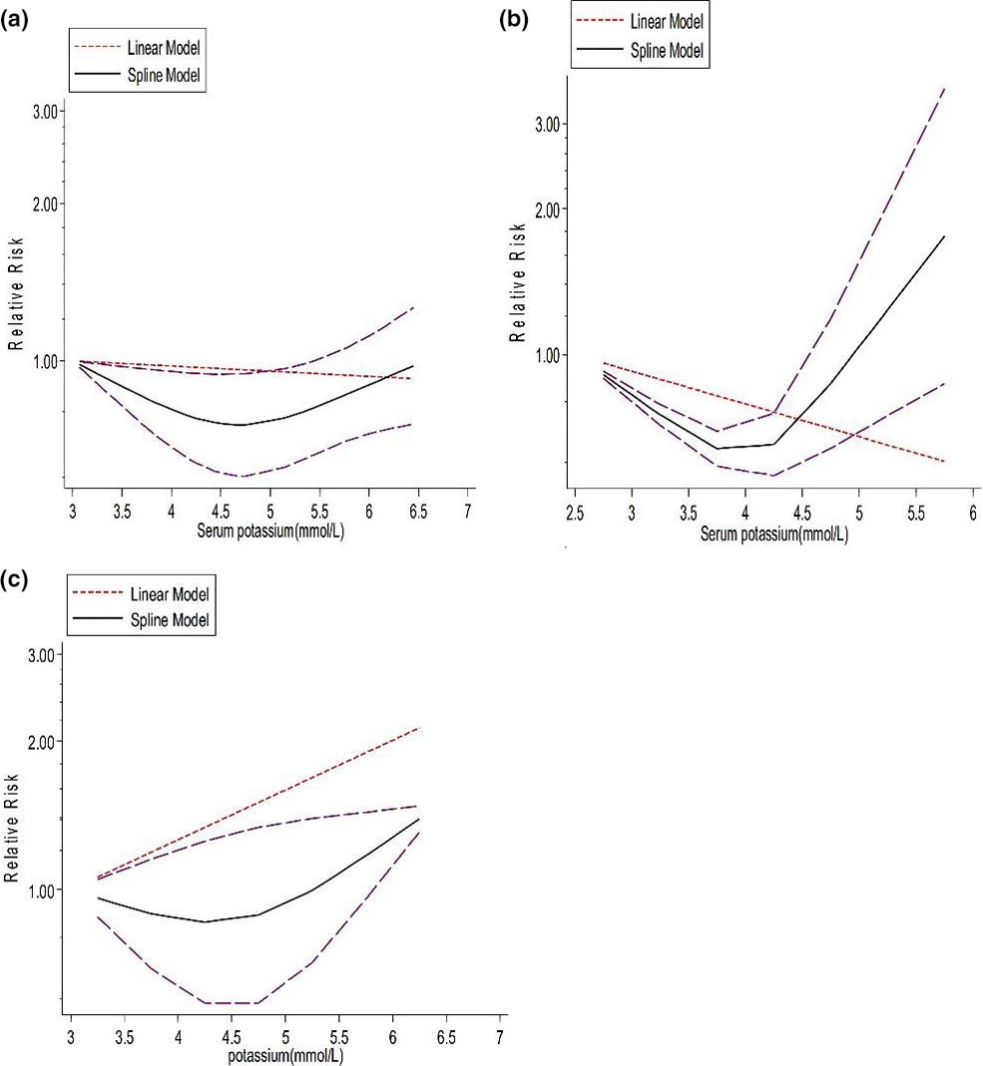
Horizontal axis shows serum potassium level and vertical axis shows OR. Solid line and long‐dash lines represent relative risk and 95% CIs. (a) Nonlinear dose–response association between serum potassium and risk of all‐cause mortality in chronic kidney disease (CKD) patients (hemodialysis patients ‐ *p* =.501). (b) Nonlinear dose–response association between serum potassium and risk of all‐cause mortality in CKD patients (peritoneal dialysis patients – *p* =.070. (c) Nonlinear dose–response association between serum potassium and risk of all‐cause mortality in CKD patients (patients with CKD without renal replacement therapy – *p* =.234)

Although we separately tested the potential curve linear associations in studies with different CKD patients, the reanalysis of data from CKD patients undergoing HD/peritoneal dialysis (PD) or CKD patients without renal replacement therapy did not result in significant associations (Figure 3). The analysis of data from CKD patients undergoing HD, we detected a flattened J‐shaped curve (*p* for nonlinearity =0.501) with an estimate in the correlation matrix of −0.022 and the estimated between studies SDs of −0.088 and 0.0432 (Prob > chi^2^ = 0.0000). Other subgroup analyses also revealed similar nonsignificant nonlinear curves in CKD patients undergoing PD (*p* for nonlinearity=0.070, estimate in the correlation matrix of −0.156, estimated between studies SDs of −0.325 and 0.012, Prob > chi^2^ < 0.0001) and CKD patients without renal replacement therapy (*P* for nonlinearity=0.234, estimate in the correlation matrix of −0.097, estimated between studies SDs of −0.258 and 0.063, Prob > chi^2^ < 0.0001).

#### 3.2.5 Linear association

Eighteen studies reported information on the association between serum potassium and risk of all‐cause mortality in patients with CKD (Einhorn et al., 2009; Flueckiger et al., 2014; Fukui et al., 2014; Heerspink et al., 2014; Hwang et al., 2011; Korgaonkar et al., 2010; Kovesdy et al., 2007; Lee et al., 2017; Li et al., 2015; Luo et al., 2016; Torlen et al., 2012; Xu et al., 2014; Zhang et al., 2016). The linear dose–response meta‐analysis showed that the risk of all‐cause mortality declined by 1% for a 0.5 mmol/L increment in serum potassium in the random‐effects model (Pooled RR: 0.99, 95% CI 0.97, 1.014, *p* = .675; Figure 3a) However, this association was not statistically significant which highlighted that the association between serum potassium and mortality is not linear. However, a high heterogeneity was detected among the studies in the linear relationship (*I* ^2^=99.9%, *P*_heterogeneity_ < .0001) (Figure 3.a).

To find potential sources of heterogeneity, subgroup analyses were performed. Subgroup analyses based on type of renal disease (CKD, HD, and PD) and age (<60 years and ≥60 years) did not reveal sources of heterogeneity. The subgroup analyses in the linear dose–response meta‐analysis revealed the following results: the linear dose–response analysis in HD patients revealed that the risk of all‐cause mortality decreased by 1% for a 0.5 mmol/L increment in serum potassium (pooled RR: 0.991, 95% CI: 0.977–1.004, *p* =.169 and heterogeneity: 99.8%, *p* =.000). In PD, the risk of all‐cause mortality decreased by 3% for a 0.5 mmol/L increment in serum potassium (pooled RR: 0.972, 95% CI: 0.930–1.017, *p* =.217 and heterogeneity: 99.4%, *p* =.000). In CKD patients without renal replacement therapy, the risk of all‐cause mortality decreased by 6% for a 0.5 mmol/L increment in serum potassium (pooled RR: 0.940, 95% CI: 0.892–0.991, *p* =.021 and heterogeneity: 96.9%, *p* <.001). We also investigated linear associations in age subgroups: age <60 years ((HR: 0.964, 95% CI: 0.930–0.999, *p* =.047 and heterogeneity: 98.7%, *p* <.0001)) and ≥60 years (HR: 1.007, 95% CI: 0.986–1.028, *p* =.521 and heterogeneity: 99.9%, *p* <.0001).

#### 3.2.6 Sensitivity analysis, subgroup analysis, and publication bias

In the sensitivity analysis, by removing each study at a time, we found out that three studies had considerable effects on the summary results. However, none of them made the heterogeneity disappear. The linear dose–response analysis showed that the risk of all‐cause mortality declined by 2% for a 0.5 mmol/L increment in serum potassium after removing the Einhorn et al.’s inpatients (pooled RR: 0.98, 95% CI 0.97, 0.99) and Einhorn et al.’s outpatients data (pooled RR: 0.98, 95% CI 0.96, 0.99, Figure 3b). After removing data from Lee et al.’s study, the risk of mortality did not change for a 0.5 mmol/L increment in serum potassium. Additionally, no publication bias was detected among the studies (Egger's regression test *p*‐value = 0.405) which also was confirmed by the funnel plot (Figure 3c).

## 4 DISCUSSION

The present dose–response meta‐analysis was included 15 prospective studies with a total number of 1,122,325 participants and 123,274 cases of all‐cause mortality. The nonlinear dose–response meta‐analysis suggested a J‐shaped association between serum potassium and the risk of all‐cause mortality in patients with CKD, with a nadir at serum potassium of 4.5 mmol/L. The risk of mortality increased when serum potassium exceeded 4.5 mmol/L with a steep slope. The subgroup analyses did not show a significant association between serum potassium and all‐cause mortality in CKD patients undergoing HD, PD or in CKD patients without renal replacement therapy. In patients over 60 years, do not follow J‐shape association which was also statistically significant. The linear analysis did not show any significant association.

Hypokalemia and hyperkalemia are commonly detected in patients with CKD. Hyperkalemia has been linked to several factors including reduced eGFR, treatment with renin–angiotensin–aldosterone system (RAAS) blockers and a cellular shift due to the presence of acidosis or diabetes (Moranne et al., 2009; Seliger et al., 2008; Williams, 1991). On the other hand, diabetes mellitus could be a potential independent predictor of hyperkalemia due to the dual role of aldosterone in governing potassium homeostasis, oxidative stress, and atherosclerosis (Fiebeler & Luft, 2005). When aldosterone or other kaliuretic factors fail to preserve the potassium homeostasis, extracellular potassium levels may increase (Gennari & Segal, 2002). In addition, it must be noted that potassium adjustment is the response of the kidneys to a high dietary potassium intake (Palmer, 1999). It has been reported that, in patients with renal insufficiency, the body can adapt to a new steady level of serum potassium which is considerably higher than the normal level (Salem et al., 1991). Other adaptive mechanisms in patients with CKD could be increased extra‐renal potassium excretion through the gut as kidney function declines (Ahmed & Weisberg, 2001). Moreover, hypokalemia has been reported to be a manifestation of hypo‐albuminemia and malnutrition, diuretic use and chronic inflammation (Kim et al., 2007; Szeto et al., 2005). In the setting of PD, lower serum potassium could be due to the stimulation of glucose absorption from the dialysate which can cause an intracellular uptake of potassium concurrent with the release of insulin (Musso, 2004). Furthermore, the renal function is better preserved in patients undergoing PD versus HD, with hypokalemia being more likely (Lang et al., 2001; Misra et al., 2001).

Our results highlight the J‐shaped association between serum potassium and mortality among CKD patients which is concordant with previous large studies cohort studies. However, in our research we pooled all the studies in a comprehensive meta‐analysis which has not performed until now. Disturbances in serum potassium could influence several leading causes of mortality. The increased mortality associated with hypokalemia may be attributed to: (a) major cardiovascular outcomes, including hypertension, ventricular arrhythmia, atrial fibrillation, stroke, heart attack as well as other risk factors (Kalantar‐Zadeh et al., 2006; Oikawa et al., 2009; Sica et al., 2002). Mounting evidence reports that elevated blood pressure is as a result of potassium deficiency *via* endothelial dysfunction (Hadi et al., 2005; He et al., 2010). Low serum potassium levels impact the myocardial resting membrane potential, enhancing the chance of ventricular arrhythmias, and sudden cardiac death (Schulman & Narins, 1990). (b) Endocrine outcomes, that is, impairment of insulin release and insulin sensitivity, resulting in hyperglycemia. Hyperglycemia is accompanied by several complications which increase mortality (Bielecka‐Dabrowa et al., 2012; Kjeldsen, 2010; Lu et al., 2009). (c) muscle‐related complications which influence the smooth muscles of the gastrointestinal tract, leading to a reduced appetite and even paralytic ileus which could be linked to malnutrition (Lu et al., 2009). Malnutrition is a key factor in the long‐term survival of these patients (Mehrotra et al., 2011; Szeto et al., 2005). Increased mortality also has been reported in patients who received high doses of thiazides related to the consequent hypokalemia (Hoes et al., 1997). Previous observations also reported increased infectious risk in PD patients probably *via* alteration of the intestinal motility. When accompanied by protein‐energy wasting, hypokalemia can impair the immunological defenses of the organism and can cause bacterial overgrowth and enhanced vulnerability to Enterobacteriaceae‐associated peritonitis (Chuang et al., 2008; Murata et al., 1993). Both hypo‐ and hyperkalemia have been associated with arrhythmias. Hyperkalemia has been linked with an increased risk of ventricular arrhythmias and increased all‐cause mortality in patients with CKD (Genovesi et al., 2009). Hyperkalemia can lead to a rapid decrease in the resting membrane potential, leading to an augmented cardiac depolarization, muscle excitability, and probable electrocardiographic changes (Mattu et al., 2000). These cardiac changes may be exacerbated in patients with kidney disorders. If accompanied by other electrolyte disturbances, the cardiac membrane potential can be affected (Michael., 2001).

Our data analysis revealed a different dose–response association at age over 60 that do not follow J‐shape association. A potential explanation could be related to the multimorbidity of patients older than 60 which puts them at risk of death regardless of their serum potassium levels. Thus, we may hypothesize that mortality in patients aged >60 is less sensitive to serum potassium changes as reported in a previous study (Georges N Nakhoul et al., 2015). The total exchangeable body potassium is 15% lower in aged subjects compared to young people. This might be related to the reduced muscle mass in the elderly (Musso & Macías‐Núñez, 2008). Aged kidneys also have a lower rate of potassium excretion due to low potassium secretion and high potassium re‐absorption (Musso et al., 2006). Similarly, excessive potassium re‐absorption may occur in the elderly (Musso et al., 2006). In addition, in the elderly, not only are plasma aldosterone levels decreased, but also kidney tubules become resistant to aldosterone. Since aldosterone is an important stimulus for the renal potassium secretion, these changes lead to a decreased excretion of potassium in the elderly (Nuñez et al., 1987). The elderly also present an impaired capacity to activate suitable responses to RAAS stimuli. This age‐related reduction in plasma renin and aldosterone may cause hyperkalemia (Yoon & Choi, 2014). Urinary potassium output is low among the elderly especially when they are treated with anti‐aldosterone agents, angiotensin‐converting enzyme inhibitors, or angiotensin II receptor blockers (Musso & Macías‐Núñez, 2008). Two markers of potassium excretion are the trans‐tubular potassium concentration gradient and the fractional excretion of potassium. Both are reportedly lower in healthy elderly subjects compared to other conditions associated with a low GFR, such as CKD (Musso et al., 2006). In conclusion, low aldosterone levels and a tendency to retain potassium, in association with old age, predispose the elderly to hyperkalemia.

The findings from the linear analysis confirm our results in the nonlinear analysis, that is, that there is a departure from linearity. In essence, an increase of 0.5 mmol/L of serum potassium could not explain the association between serum potassium levels and mortality, since, as we demonstrated, the association is J‐shaped.

Several CKD clinical handbooks have reported this J‐shaped association. For patients undergoing dialysis, a nadir of 5–5.5 mmol/L has been reported. The highest mortality has been linked to serum potassium concentrations higher than 6.5 mmol/L and lower than 4 mmol/L. It has been shown that malnourished patients may have low predialysis serum potassium levels. Therefore, the dialysate potassium level could be monitored to prevent hypokalemia. In addition, the diet must be monitored in terms of dietary potassium intake. Moreover, when patients stop ingesting high potassium diets for any reason, the potassium in the dialysate must be checked again.

This study also has several shortcomings and strength that must be mentioned. The first limitation could be the difference in follow‐up durations that might contribute to the inter‐study heterogeneity. Although potential confounding factors are possibly limited by excluding studies which reported unadjusted HRs, our results could have been influenced by confounding factors which existed in the original cohort studies. In addition, we did not register the protocol of the current study on PROSPERO registry system due to the delay in processing the submitted protocols for studies outside the UK. This lack of registration might be a source of bias for this review. However, this review and meta‐analysis was designed and performed according to the Cochrane guidelines.

The strengths of our study could be the dose–response meta‐analysis aimed at a better understanding of the strong dose‐dependent association between serum potassium and all‐cause mortality among CKD patients. This association enabled us to show the potential optimal levels of serum potassium in CKD patients. Also, we conveniently showed that the shape and strength of the association between serum potassium and all‐cause mortality in patients with CKD is strongly influenced by age. There was a clear difference in older patients (>60 years). The other strength is that, since we investigated mortality, only cohorts were included in the meta‐analysis. Thus, we could show a causality relationship. Thirdly, the sample size of the meta‐analysis was considerable, with more than one million participants, enabling us to achieve a relatively strong statistical inference.

## 5 CONCLUSION

The present meta‐analysis provides supportive evidence regarding the J‐shape association between serum potassium and all‐cause mortality among CKD patients. The nadir was detected at 4.5 mmol/L. Potassium values lower or higher than this level were associated with increased mortality among CKD patients. This association was, however, different in patients above 60 years.

## CONFLICTS OF INTEREST

The authors declare that there are no conflicts of interest regarding the publication of this paper.

## AUTHOR CONTRIBUTIONS

N.S and Sh.T designed research and contributed to the conception of the project, development of overall research plan, and study oversight. All authors drafted the manuscript. N.S analyzed and interpreted the data. A.F and B.H contributed to the final revision of the manuscript. All authors critically revised the manuscript for important intellectual content and approved it for submission.

## ETHICAL APPROVAL

Ethical Review: This study does not involve any human or animal testing. Ethics approval was not required for this research.

## Data Availability

The data from this study are available upon request from the corresponding author.
